# 3D printer platform and conductance feedback loop for automated imaging of uneven surfaces by liquid microjunction‐surface sampling probe mass spectrometry

**DOI:** 10.1002/rcm.9492

**Published:** 2023-02-26

**Authors:** Matthias Hermann, Haidy Metwally, Jian Yu, Rachael Smith, Hailey Tomm, Martin Kaufmann, Kevin Y. M. Ren, Chang Liu, Yves LeBlanc, Thomas R. Covey, Avena C. Ross, Richard D. Oleschuk

**Affiliations:** ^1^ Department of Chemistry Queen's University Kingston Ontario Canada; ^2^ Department of Surgery Queen's University Kingston Ontario Canada; ^3^ Department of Pathology Queen's University Kingston Ontario Canada; ^4^ SCIEX Concord Ontario Canada

## Abstract

**Rationale:**

Molecular imaging of samples using mass spectrometric techniques, such as matrix‐assisted laser desorption ionization or desorption electrospray ionization, requires the sample surface to be even/flat and sliced into thin sections (*c*. 10 μm). Furthermore, sample preparation steps can alter the analyte composition of the sample. The liquid microjunction‐surface sampling probe (LMJ‐SSP) is a robust sampling interface that enables surface profiling with minimal sample preparation. In conjunction with a conductance feedback system, the LMJ‐SSP can be used to automatically sample uneven specimens.

**Methods:**

A sampling stage was built with a modified 3D printer where the LMJ‐SSP is attached to the printing head. This setup can scan across flat and even surfaces in a predefined pattern (“static sampling mode”). Uneven samples are automatically probed in “conductance sampling mode” where an electric potential is applied and measured at the probe. When the probe contacts the electrically grounded sample, the potential at the probe drops, which is used as a feedback signal to determine the optimal position of the probe for sampling each location.

**Results:**

The applicability of the probe/sensing system was demonstrated by first examining the strawberry tissue using the “static sampling mode.” Second, porcine tissue samples were profiled using the “conductance sampling mode.” With minimal sample preparation, an area of 11 × 15 mm was profiled in less than 2 h. From the obtained results, adipose areas could be distinguished from non‐adipose parts. The versatility of the approach was further demonstrated by directly sampling the bacteria colonies on agar and resected human kidney (intratumoral hemorrhage) specimens with thicknesses ranging from 1 to 4 mm.

**Conclusion:**

The LMJ‐SSP in conjunction with a conductive feedback system is a powerful tool that allows for fast, reproducible, and automated assessment of uneven surfaces with minimal sample preparation. This setup could be used for perioperative assessment of tissue samples, food screening, and natural product discovery, among others.

## 1 INTRODUCTION

Mass spectrometry imaging (MSI) techniques are an important tool for analyzing various materials, including biological samples such as plants, bacteria, and mammalian tissue samples. The techniques combine the high sensitivity and specificity of MS with spatial information by correlating recorded mass spectra to different locations across the sample.^1^, ^2^ The spatial distribution of analytes across a sample can then be illustrated using heatmaps where the color temperature indicates ion(s) intensity. Standard MSI techniques are matrix‐assisted laser desorption ionization (MALDI),^3^, ^4^, ^5^ desorption electrospray ionization (DESI),^6^ laser ablation electrospray ionization (LAESI),^7^ and direct analysis in real time (DART).^8^, ^9^ These imaging methods have outstanding spatial resolutions of tens of micrometers but require more elaborate sample preparation, such as embedding the sample into resins to enable accurate slicing to even thicknesses of several micrometers. Other common sample preparation steps are washing and removal of embedding material, and for MALDI, the surface application of matrix materials to enhance ionization.^10^, ^11^ The resulting sample preparation steps are time consuming and costly; however, efforts are showing that further efficiency is possible. Furthermore, MALDI requires samples to be dry for analysis due to the analysis under vacuum. Compared to that, DESI allows for analysis under ambient conditions and can tolerate uneven surfaces up to 1 mm; however, a consistent humidity is necessary for reproducible results and experienced personnel for parameter tuning and setup. Even more critical is that sample handling can alter the presence, amount, and distribution of analytes across the sample and lead to strong background noise.^12^, ^13^ On the other end of the spectrum is the MassSpec Pen, for rapid sampling of surfaces without sample preparation. Upon contact with a tissue, a water droplet for analyte extraction is formed in the MassSpec Pen. As a handheld device with a spatial resolution of 2.7 mm, it allows for quick assessment of tissue samples.^14^, ^15^, ^16^

**FIGURE 1:**
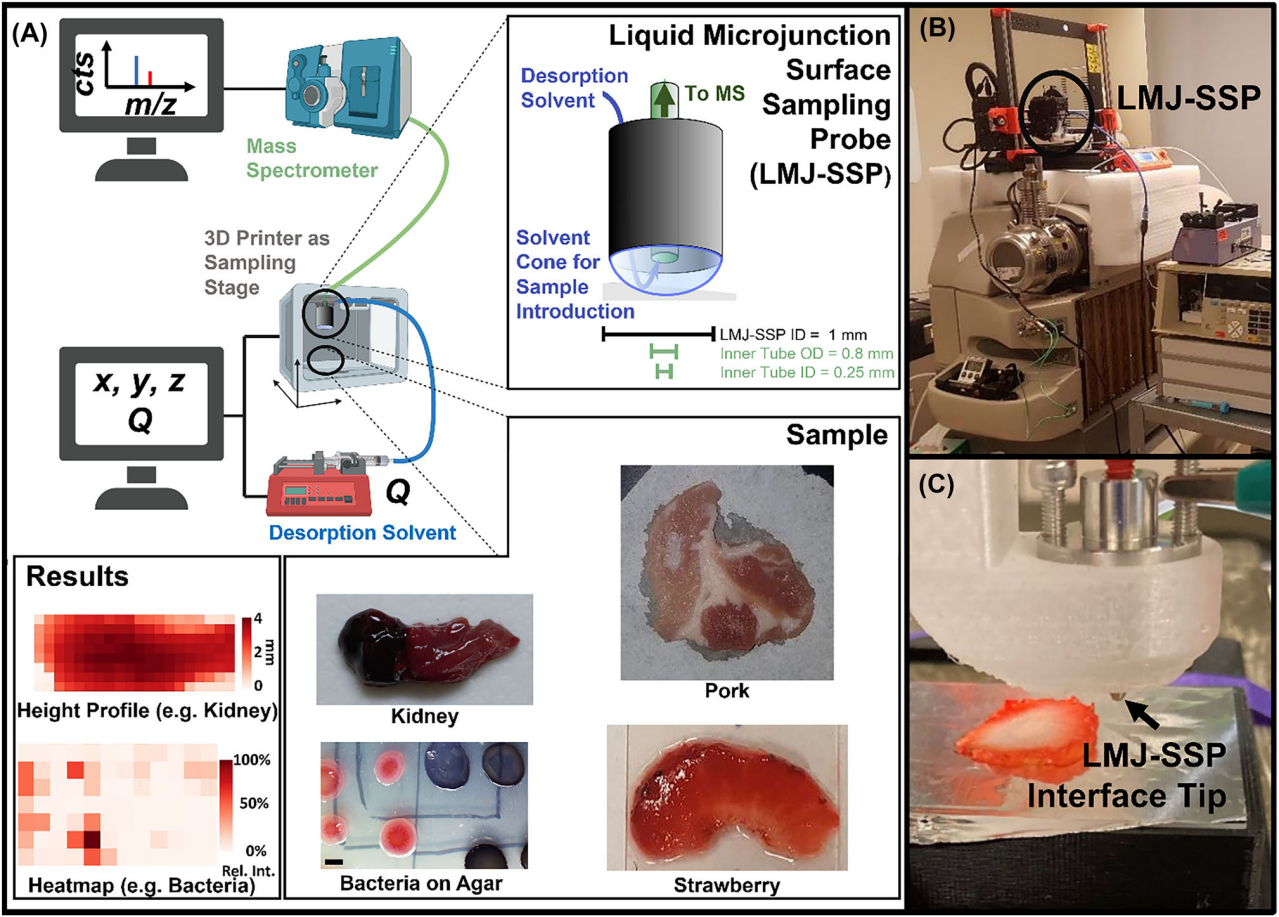
A, Schematic diagram of the liquid microjunction‐surface sampling probe (LMJ‐SSP). The LMJ‐SSP consists of two open‐ended concentric tubes. A desorption solvent is delivered via a syringe pump between these tubes and then drawn into the inner tube that is connected to the mass spectrometer. A variety of samples, such as liver and pork tissue, bacterial colonies on agar, and strawberry samples in some cases with no sample preparation, can be directly interrogated by the LMJ‐SSP. Heatmaps that show the spatial distribution of analytes and height profiles can be obtained. B, Setup for automated imaging using a modified 3D printer. The LMJ‐SSP is attached to the printer head of a 3D printer that sits on top of the mass spectrometer. C, Image of the LMJ‐SSP in a 3D printed holder sampling an uneven strawberry slice with a thickness of *c*. 3 mm. [Color figure can be viewed at wileyonlinelibrary.com]

The liquid microjunction‐surface sampling probe (LMJ‐SSP)^17^, ^18^, ^19^, ^20^ falls in between imaging techniques with micrometer spatial resolution (MALDI, DESI, and LAESI) and the MassSpec Pen for single‐point assessment of millimeter‐wide sampling spots. With a sampling spot size of 1 mm, its ability to analyze samples without sample preparation, and as an ambient sampling technique, it can be used not only for single‐point assessment but also for imaging and profiling. Possible applications are perioperative diagnosis, natural product discovery, and quality control. The LMJ‐SSP consists of two open‐ended concentric tubes through which a desorption solvent is pumped (Figure 1A). The inner tube is connected to a Venturi that draws the liquid that has exited the outer tube from the probe tip. The liquid is then introduced to the mass spectrometer through an electrospray interface. Due to the surface tension of the desorption solvent, a liquid dome is formed at the end of the concentric tubes.^21^, ^22^, ^23^ Specimens placed either into or those touching the liquid dome are sampled and delivered to the MS within seconds. This method of introduction requires minimal sample preparation. Despite the robustness of the LMJ‐SSP and the ability to analyze samples under ambient conditions, the sample surface still needs to be flat to assure reproducible contact between the probe and the sample. Otherwise, the probe may not touch the surface, or the probe can be pushed into the surface. Approaches to enable reproducible sampling of uneven surfaces by MSI included the assessment of surface profiles using real‐time camera image analysis,^24^, ^25^ 3D laser scans,^26^ and laser‐point distance sensors.^27^ We propose an alternative approach that utilizes an electrical conductive feedback system to enable sampling of uneven surfaces with height differences of several millimeters but could be extended to samples with much larger variations. The probe is lowered toward each sampling spot until the conductance feedback signal is triggered.

Reproducible manipulation of the probe relative to the sample can be achieved using a programmable gantry/sampling stage. However, commercially available solutions are often expensive and have limited customizability. The emerging role of 3D printing in laboratories and specifically for mass spectrometry application has been reported^28^, ^29^, ^30^, ^31^ and was implemented using a modified 3D printer (Figures 1B and 1C). Due to the limited flexibility of 3D printer movements, surfaces can be sampled from only one direction. If the probe and the conductance feedback device are controlled by a robotic arm (e.g., 6‐axis), more complicated shaped objects may be analyzed from different directions. However, in both cases, the distribution of analytes is analyzed only on the surface and across a 2D plane. For 3D images, the impact of analytes at different depths relative to the surface would need to be probed.

## 2 EXPERIMENTAL

### 2.1 Materials and samples

All solvents were analytical grade and purchased from Sigma Aldrich (St Louis, MO, USA). Strawberry and porcine samples were purchased from a local grocery store and sliced using a knife. Bacteria colonies of *Pseudoalteromonas rubra DSM6842* and *Pseudoalteromonas tunicata DSM14096* were streaked on agar plates containing Difco Marine Media 2216 with 1% (w/v) agar and incubated overnight at 30°C before imaging.

### 2.2 Kidney specimen

A human kidney sample was obtained by the Department of Pathology for analysis using the LMJ‐SSP, as approved by Queen's University Health Sciences Research Ethics Board, permit no. 6018391. The kidney specimen contained areas of clear renal cell carcinoma that exhibited intratumoral hemorrhage adjacent to non‐neoplastic parenchyma. The specimen was stored at −80°C and thawed before analysis using the LMJ‐SSP. This study was conducted in accordance with the principles outlined in the Declaration of Helsinki for the use of human tissue.

### 2.3 Liquid microjunction‐surface sampling probe

Samples were analyzed using a research‐grade open port interface (OPI) provided by SCIEX (Concord, Canada). The OPI is an LMJ‐SSP consisting of two open‐ended concentric tubes. A stainless‐steel body with a circular opening (d = 1 mm) acts as the outer tube. A peek tube (1/32“ OD × 0.010” ID, IDEX, Oak Harbor, WA, USA) connected to the mass spectrometer's ionization source is used as the inner tube.

The inner tube is inserted into the stainless‐steel body, and its open end is aligned with the opening of the stainless‐steel enclosure. For all samples, the desorption solvent was delivered between the inner tube and the enclosure with a syringe pump (Chemxy Fusion 100, Chemyx Inc., Stafford, TX, USA).

### 2.4 Mass spectrometer

Strawberry samples were analyzed using a 3200 triple quadrupole mass spectrometer (Sciex), porcine and bacterial samples using a 4500 triple quadrupole mass spectrometer (Sciex), and kidney samples using a 5500 triple quadrupole mass spectrometer (Sciex). All spectra were recorded in Q1 full scan, positive ion mode with MeOH:H_2_O:formic acid (90:9.95:0.05 v/v %) as the desorption solvent. Three mass spectrometers were used in different locations with specific biohazard classifications. Furthermore, the use of the platform with different instrumentation highlights portability and compatibility with different mass spectrometer models.

### 2.5 Sampling

**FIGURE 2:**
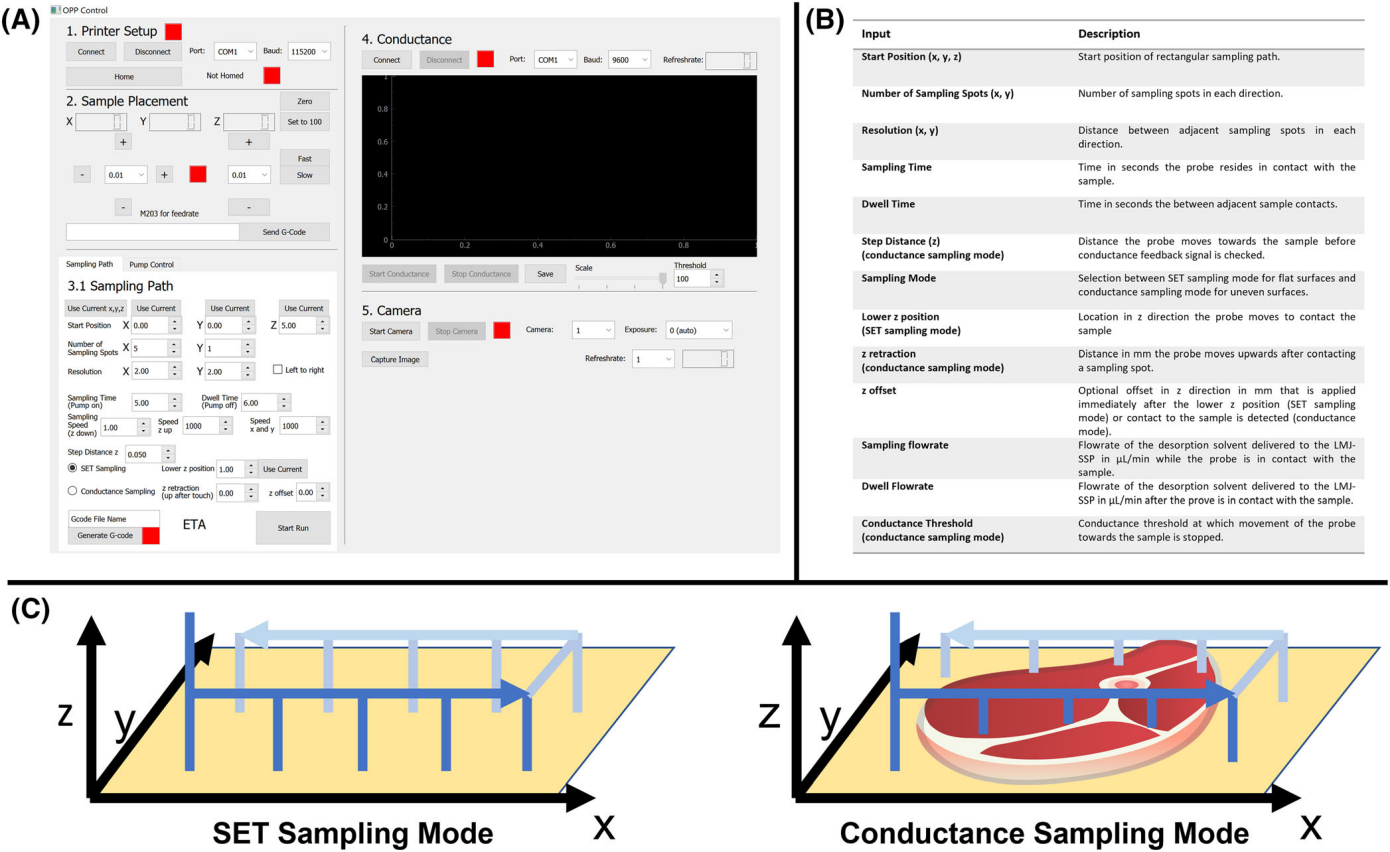
A, Software interface used to control the position and sampling path of the liquid microjunction‐surface sampling probe (LMJ‐SSP) that is attached to a modified 3D printer. B, Overview of input parameters to control the movement of the LMJ‐SSP relative to a sample. C, Schematics showing the difference between the “static sampling mode” where the probe moves to the same z‐height for each sampling spot and the “conductance sampling mode” where the probe moves toward the sample until a conductance feedback loop is triggered, leading to different z‐heights. [Color figure can be viewed at wileyonlinelibrary.com]

A modified 3D printer (Prusa, i3‐MK3, Prague, Czech) was used as a gantry to control the movement of the LMJ‐SSP relative to the sample. The sample was placed on the printing bed and the LMJ‐SSP to the printer head with a 3D printed holder (Figure 1C). The 3D printer was controlled through an in‐house written program (Python 3.1 and PyQtDesigner; Figure 2A) to control the movement of the LMJ‐SSP relative to the sample by sending G‐code commands via a serial USB connection to the 3D printer. The software generates G‐code commands based on inputs as shown in Figure 2B to sequentially move the probe toward the sampling spots that are arranged in a rectangular pattern across the surface. Once a sampling spot is reached, the probe resides in this position for the sampling time (Figure 2B “sampling time”). Following that, the probe is raised above the sampling spot where it resides for the “dwell time” (Figure 2B), after which the probe moves above the next sampling spot. The probe then approaches this sampling spot by moving the printer head toward the sample in z‐direction until the contact is achieved. The procedure was repeated for the remaining sampling spots. Sampling spots can be approached in “static sampling mode” or in “conductance sampling mode” (Figure 2C). In the static sampling mode, the probe is moved to the same z‐height for each sampling spot, allowing one to analyze flat and leveled surfaces, such as sessile droplet arrays on surface energy traps^32^, ^33^, ^34^ or microtomed tissue sample. The conductance sampling mode can be utilized for automated sampling of uneven and unleveled samples. In this mode, the probe moves stepwise toward the sample (Figure 2B; “step distance,” e.g., 50 μm). After each step, a feedback signal is used to check for contact between the probe and the sample. A triggered feedback signal indicates that the sampling spot is reached, and the movement of the probe in z‐direction toward the sample is stopped. The feedback signal is based on the voltage measured at the sample. A voltage of 5 V is applied to the sample through an electrically conductive sample bed (e.g., tin foil or an indium tin oxide‐coated glass slide) and is measured via an analog input pin of a microcontroller (Arduino Nano). An analog‐to‐digital converter within the microcontroller converts the voltage to a digital 10‐bit value (*sensorValue*) between 0 (0 V) and 1023 (5 V), which is sent to the program that controls the movement of the LMJ‐SSP. Once the LMJ‐SSP, whose body is grounded, comes into contact with the sample, the voltage applied to the sample is drawn to ground, leading to a drop in the measured voltage triggering the feedback signal. The software interface plots the *relativeConductance* = 1023 – *sensorValue* (Figure 2A). Therefore, *relativeConductance* = 0 when there is no contact between the sample and probe. When there is contact between the probe and the sample, *relativeConductance* > 0 and peaks are plotted. This reversion of the digital values makes the plot more intuitive and user‐friendly. A threshold value for the *relativeConductance* can be defined above which the feedback signal is triggered (Figure 2B; “threshold”). This threshold value can be adjusted depending on the electrical conductivity of the sample and the desorption solvent employed. An array of *relativeConductance* values with its respective times and relative position of the probe in *x*‐, *y*‐, and *z*‐directions is recorded by the program and can be exported as *.csv file. This *.csv file can be used for further data analysis steps to generate height profiles and for peak alignment.

### 2.6 Data analysis

**FIGURE 3:**
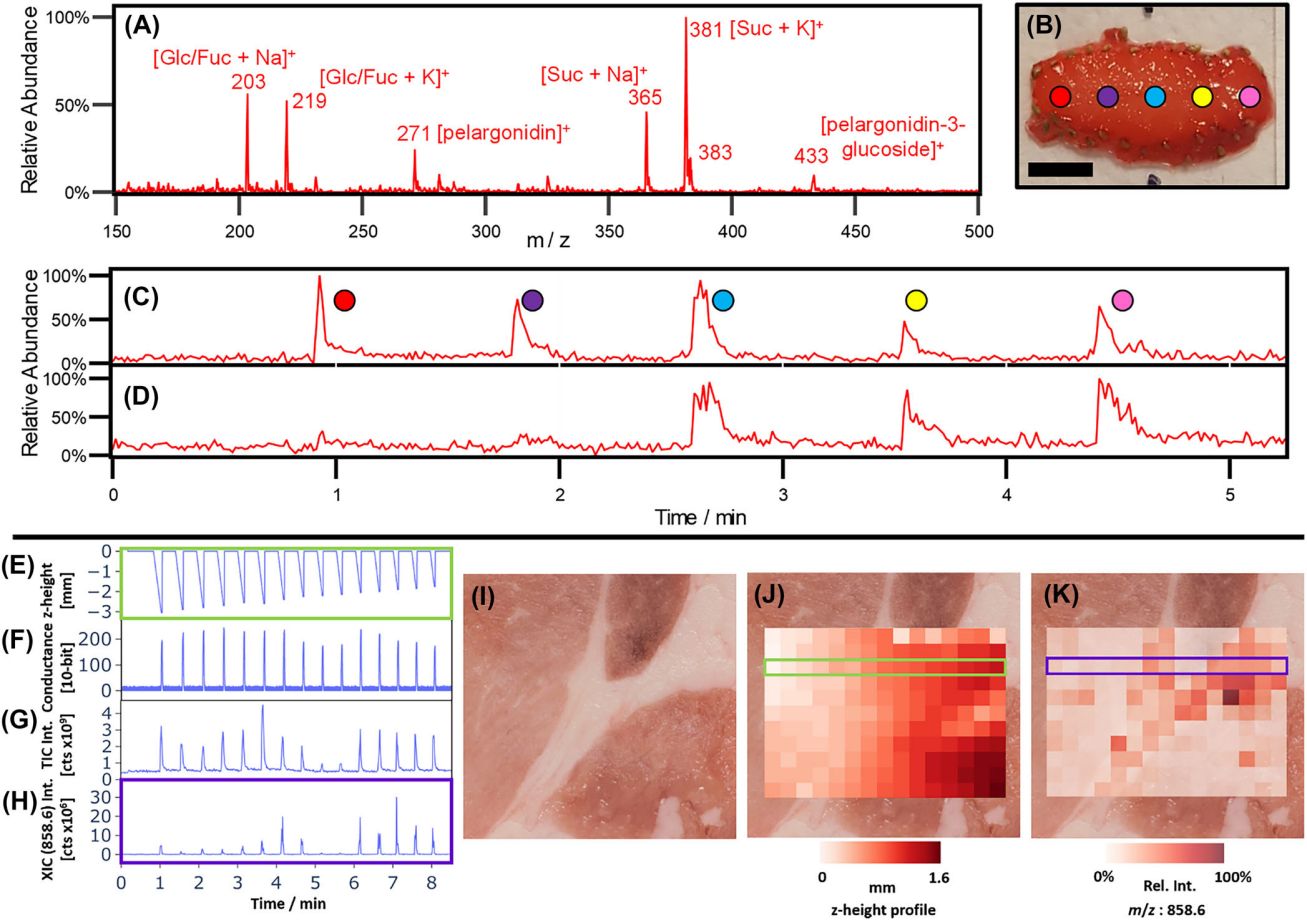
Results from scanning with a liquid microjunction‐surface sampling probe (LMJ‐SSP) across a strawberry slice. A, Spectrum of a strawberry slice where characteristic peaks are labeled. B, Optical image of the strawberry sample that was manually sampled at five points along a line. The scale bar is 5 mm. C, Total ion current (TIC) for a range of *m/z* = 150–500, where the peaks of each sampling spot are color‐coded using the labels from B. D, Extracted ion current (XIC) of the same run for *m/z* = 195 ± 1 shows where caffeine was detected. Results for porcine tissue. E, The plot shows the z‐position of the probe relative to its starting position (z = 0) for one horizontal line (see green frame in Panel J) during an automated run utilizing the conductive feedback system. F, Conductance trace recorded using the analog‐to‐digital converter of the microcontroller. Peaks indicate the times at which the probe contacts the sample. G, Total ion current. H, Extracted ion current for *m/z* = 858.6 ± 1 for one horizontal line (see purple frame in Panel K). I, Image of the analyzed porcine tissue. J, Overlay of a picture of the porcine tissue and the z‐height profile extracted from the conductive feedback system. The maximum height difference is 1.6 mm. K, Overlay with a heatmap of *m/z* = 858.6. Higher concentrations can be seen in the adipose tissue that separates the areas of red tissue. [Color figure can be viewed at wileyonlinelibrary.com]

Mass spectra were acquired in *.wiff format through Analyst (1.6.3 SCIEX, Concord, Canada). For data analysis, *.wiff files were converted to *.mzml files using Proteowizard^35^ and then processed in a Python script using the pymzml module.^36^ The script also imports *.csv files generated by OPI scan that contain recorded *conductanceValues* and positional data with its respective timestamps throughout each experiment (vide supra). Imported *.mzml files contain all the spectra acquired throughout the experiment. However, several spectra are based on the composition of desorption solvent that was not in contact with the sample. Therefore, only the spectra that show the composition of desorption solvent that was in contact with the sample are of interest and need to be extracted and assigned to their respective spatial location. Different approaches can be used to identify the spectra of interest. The easiest way is through the intensities of the recorded mass spectra themselves. The total ion current (TIC) of each spectrum is plotted against their respective times. Spectra of interest appear as peaks in the TIC when analytes are picked up/extracted and detected during sampling. Figure 3C shows a TIC of 15 sampling spots. Even the lowest peaks (#9 and #10) can be distinguished from the baseline (e.g., by peak picking). Alternatively, an *m/z* value detected across all sampling spots can be used to find the times corresponding to spectra of interest (e.g., by the extracted ion current [XIC]). However, this approach falls short for experiments where the amount of extracted and detected ions on sampling spots is low, for example, due to low analyte concentration or a narrowly measured *m/z* range. For reliable identification of spectra of interest independent from the recorded mass spectra, the measured *conductanceValues* (Figure 3B) can be used. Recorded *conductanceValues* precisely trace the times at which the probe contacts the surface. From these contact times, the times at which mass spectra of interest appear in the MS chronogram can be extrapolated using the delay between sample contact and mass detection (*t*_offset_). All spectra recorded in a defined time window around that peak time are extracted (e.g., ±4 s). The length of this time window can be adjusted based on factors such as the sampling time and the amount of analyte extracted and detected. Finally, all spectra of the same time window are averaged, leading to one mass spectrum for each sampling spot (pixel). Extracting a given *m/z* range from each averaged spectrum (XIC) and plotting them based on the location of their sampling spot allow one to create heatmaps that show the spatial distribution of that *m/z* range across the sample surface (Figure 1A; “height profile”). Furthermore, a surface profile can be generated when the height of the probe (z position) during sample contact (peak of conductance trace) is assigned to each pixel (Figure 1A; “heatmap”).

## 3 RESULTS AND DISCUSSION

To test the utility of the LMJ‐SSP for direct analysis of surfaces, we analyzed several samples with surface topologies that are challenging to profile without extensive sample preparation. First, caffeine‐spiked (1 mg/ml) strawberry samples (Figures 3A–3D) were analyzed by manually adjusting the 3D printer chassis and moving the LMJ‐SSP relative to the sample, and then porcine samples were profiled using the conductive feedback system (Figures 3E–3J). Finally, the versatility of the conductive feedback system was demonstrated by sampling bacterial colonies directly from agar plates and by analyzing a human kidney sample containing renal cell carcinoma with intratumoral hemorrhage with a thickness that varies from 1 to 4 mm (Figure 1A). Heatmaps were produced showing the distribution of analytes (e.g., lipids in a porcine sample) across the tissue, enabling one to distinguish between tissue types (e.g., adipose and muscle). In addition to ion intensity profiles, the platform allows one to export the z‐heights at which the probe contacts the surface to generate height contours of the specimen. Due to the low currents and short contact times, no sample and signal alterations were observed when comparing runs with and without the conductance feedback mode.

### 3.1 Caffeine‐spiked strawberry (manual sampling)

3D printers, particularly fused deposition modeling varieties, have become more affordable and potentially offer a facile way of automatically controlling the movement of surface sampling. The LMJ‐SSP was aligned above the desired sampling location and moved toward the sample by manually controlling the modified 3D printer head. Once the extraction solvent wets the sample surface, the sampling time starts. After 6 s, the LMJ‐SSP probe was manually raised and aligned above the following sampling location, and the process was repeated (Figure S1 and Video S1 [supporting information]). Figure 3B shows the five sampling locations. Figure 3C shows the associated TIC for *m/z* = 150–500. The peaks for the five sampling locations are identifiable and color‐coded based on their positions on the strawberry slice (Figure 3B). The distribution of caffeine on the sample can be reconstructed using the XIC, as shown in Figure 3D (*m/z* = 195 ± 1). Although the first two sampling locations (red and purple) do not show a peak, the other three (blue, yellow, and pink) indicate the presence of caffeine. The procedure of manually moving the LMJ‐SSP was slow and tedious because each sampling location needed to be slowly approached from above until the probe was in contact with the sample. Moving the probe too fast or without full attention toward the sample can result in the probe being pushed into the sample, leading to flow interruption and potential probe clogging. In addition, the time the probe is in contact with the sample is difficult to control when the movement is manually manipulated.

### 3.2 Lipid heatmap of pork tissue sample (automated sampling mode)

An automated feedback system based on conductance measurements as described earlier was used in variable z‐height mode to create a heatmap of the lipid distribution on a porcine tissue sample (Figures 3I–3K). An area of 15 × 11 mm was profiled with a resolution of 1 mm. This is the lowest resolution that can be achieved without oversampling and is determined by the size of the LMJ‐SSP opening. Prior to analysis, the sample was rinsed with water and placed on the printing bed without leveling the sample surface. The recorded z‐height profile in Figure 3J shows that the height difference between the lowest part of the sample (top left [white/light red]) and the highest part of the sample (bottom right [dark red]) was 1.6 mm. The profiled area consisted of three “islands” of red tissue separated by adipose tissue. Figure 3K shows an overlay of a sample picture with the measured distribution of *m/z* = 858.6 ± 1. It is suggested that this signal corresponds to a phosphocholine lipids (e.g., PC(20:2/22:6), PC(20:4/22:4)), which have this *m/z* value in their protonated form.

In variable/dynamic sampling mode, a trace of the measured conductance feedback and the *z*‐height is recorded besides the mass spectral data (Figure 3J). Therefore, mass spectra can be precisely assigned to their respective spatial locations even when the amount of detected analyte for a sampling spot is low. Furthermore, the sample does not need to have an even surface or to be leveled before analysis. Utilizing this automated sampling approach, a sample surface can be profiled with 161 sampling spots in less than 2 h. This was considered to be an acceptable compromise between spatial resolution and profiling duration. The applied spatial resolution of 1 mm was determined by the diameter of the LMJ‐SSP and could be improved by modification of the probe. However, an improved resolution would lead to an extended sampling time for the same area. In this study, it took about 40 s per sampling spot. This time includes 5 s of sampling time and 35 s of probe alignment (raising the probe, lateral movement above the next sampling spot, and lowering of the probe toward the sample). Most of the time is lost when the probe is lowered toward the sample. This process is slow to ensure accurate contact between probe and the samples and allows for extensive rinsing, but could be sped up by combining the conductive feedback system with other distance measurement techniques (e.g., laser scanning).

Due to the constant exchange of solvent in the LMJ‐SSP, residual analyte traces at the probe are continuously removed, which can be seen in the TIC (Figures 3C and 3G) and XIC (Figures 3D and 3H) traces. The reproducibility of the conductive feedback positioning was evaluated by approaching the same sampling spot multiple times. For a solid surface such as tin foil, the standard deviation in *z*‐direction at which contact was detected was 27 μm (five replicates). For a soft tissue sample (strawberry), the deviation was 55 μm (five replicates). These inaccuracies are below and at the accuracy of the stepper motors used in the platform, which is in the range of 50 to 150 μm.^31^

### 3.3 Sampling of uneven surfaces (automated sampling mode)

Using the automated sampling approach, uneven surfaces were analyzed. Bacterial colonies protruding *c*. 1 mm above the otherwise flat agar substrate were interrogated directly without prior sample preparation. The heatmap in Figure 1A shows the distribution of prodigiosin (*m/z* = 324.4; Figure S2 [supporting information]), a characteristic molecule from *P. rubra* that correlates with the location of *P. rubra* colonies. Furthermore, it was shown that a kidney sample with a highly variable thickness reaching up to 4 mm (see Figure 1A and Figure S3 [supporting information]) could also be profiled by the automated sampling approach.

## 4 CONCLUSION

The LMJ‐SSP is a sampling interface that allows for rapid assessment of surfaces using MS. However, systematic analysis of surfaces (e.g., profiling/imaging) can be tedious if done manually. A sampling stage is presented that allows for automated assessment of surfaces using the LMJ‐SSP. The stage is based on a modified 3D printer where the probe is attached to the printer head and the sample placed on the printing bed. This allows one to analyze samples, such as tissue, without sectioning or slicing when the surface is even. A conductance feedback system is presented to simplify the sampling procedure further and allow for analysis of surfaces that are not even or not leveled. Specimens with significant height differences (>1 mm) could be automatically profiled using the LMJ‐SSP without sample preparation.

### PEER REVIEW

The peer review history for this article is available at https://publons.com/publon/10.1002/rcm.9492.

## Supporting information


**FIGURE S1.** Top left, plot of *relativeConductance* against time in ms. Main frame, liquid microjunction‐surface sampling probe (LMJ‐SSP) automatically sampling a surface in conductance sampling mode
**FIGURE S2.** Mass spectrum of a sampling spot from *Pseudoalteromonas rubra* DSM6842 showing prodigiosin at *m/z* = 324.4
**FIGURE S3.** Mass spectrum of a sampling spot from an intratumoral hemorrhage kidney specimen showing heme B at *m/z* = 324.4 and a protein envelope above *m/z* = 630

**VIDEO S1.** Supporting information

## Data Availability

Data available on request from the authors‐ The data that support the findings of this study are available from the corresponding author upon reasonable request.
