# Supplementary material for: 3D printer platform and conductance feedback loop for automated imaging of uneven surfaces by liquid microjunction‐surface sampling probe mass spectrometry
Source: Rapid Commun Mass Spectrom. 2023 Feb 26;39(Suppl 1):e9492. doi: 10.1002/rcm.9492 (PMC12062779; doi:10.1002/rcm.9492)
Supplement: Supplementary file 1 — FIGURE S1. Top left, plot of relativeConductance against time in ms. Main frame, liquid microjunction‐surface sampling probe (LMJ‐SSP) automatically sampling a surface in conductance sampling mode FIGURE S2. Mass spectrum of a sampling spot from Pseudoalteromonas rubra DSM6842 showing prodigiosin at m/z = 324.4 FIGURE S3. Mass spectrum of a sampling spot from an intratumoral hemorrhage kidney specimen showing heme B at m/z = 324.4 and a protein envelope above m/z = 630 [file RCM-39-e9492-s001.docx]

3 D Printer Platform and Conductance Feedback Loop for Automated Imaging of Uneven Surfaces by LMJ-SSP Mass Spectrometry

M. Hermann^1^, H. Metwally^1^, J. Yu^1^, R. Smith^1^, H. Tomm^1^, M.Kaufmann^2^, K. Y. M. Ren^3^,

C. Liu^4^, Y. LeBlanc^4^, T. R. Covey^4^, A. C. Ross^1^, and R. D. Oleschuk^1^*

^1^Department of Chemistry, Queen's University, Kingston, Ontario K7L 3N6, Canada

^2^ Department of Surgery, Queen's University, Kingston, Ontario K7L 3N6, Canada

^3^ Department of Pathology, Queen's University, Kingston, Ontario K7L 3N6, Canada

^4^ SCIEX, 71 Four Valley Drive, Concord, Ontario L4K 4 V8, Canada


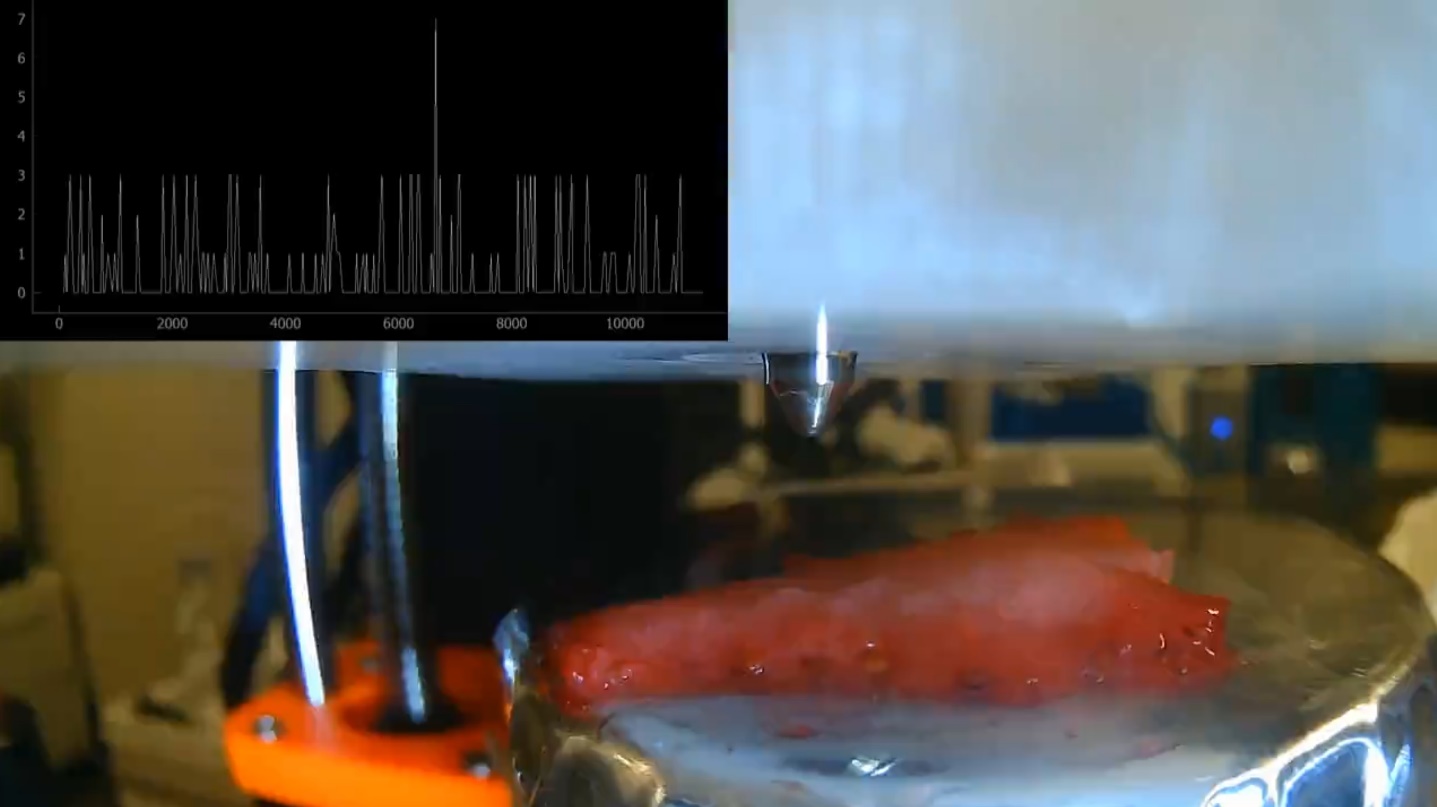
**S1:** Top left: Plot of *relativeConductance* against time in ms. Main frame: LMJ-SPP automatically sampling a surface in conductance sampling mode.

**
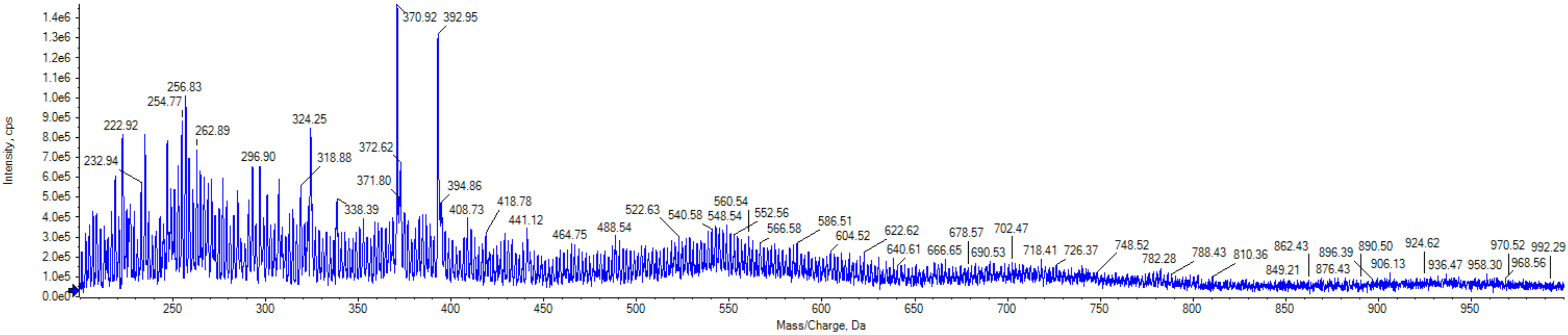
**

**S2:** Mass spectrum of a sampling spot from *Pseudoalteromonas rubra* DSM6842 showing prodigiosin at *m/z* = 324.4.


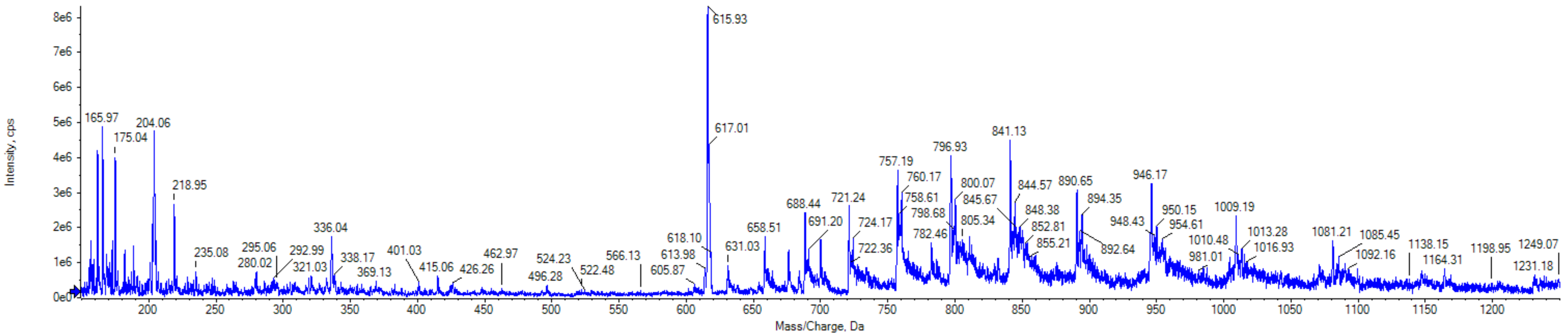


**S3:** Mass spectrum of a sampling spot from a intratumoral hemorrhage kidney specimen showing heme B at *m/z* = 324.4 and a protein envelope above *m/z* = 630.
